# Medical Hydrology

**Published:** 1914-06-06

**Authors:** R. Fortesque Fox

**Affiliations:** 36 Devonshire Place, W.


*To the Editor of* THE HOSPITAL.

### Medical Hydrology.

*To the Editor of* THE HOSPITAL.

SIR,—My attention has been called to a review of my recent work on "Medical Hydrology" in your columns. Whilst not denying the action of waters and baths in disease, the writer justly deprecates the obscurity and exaggeration of language in which the claims of this (as well as other branches of treatment) are sometimes set forth. In that opinion I cordially agree. Moreover, whilst he is kind enough to allow—for which I am duly grateful—that I am not the worst kind of offender in this respect, I am, it appears, more or less guilty. In proof whereof he quotes no words of mine, but a sentence from Professor Winternitz cited by me. Torn from its context, I admit that the statement quoted by your reviewer is startling, and I think it would have been fair to have given the whole (p. 35):

"The *modus operandi* of baths appears to be mainly upon the nervous end-organs in the skin, and through them upon the central nervous system. 'We are in a position,' says Winternitz, 'by means of hydrotherapy, to heighten innervation, to lower, abolish or alter it; and this at the point of application in the central organs and by reflection in the most varied motor and vasomotor tracts.' He adds: '*Thermal stimulations are therefore indicated when innervation requires to be strengthened, suspended or altered.*'"

The statement (italicised), at which the surgeon and the bacteriologist are supposed to laugh, sums up a mass of experimental work upon the action of heat and cold on the circulation, metabolism and nerve centres. Unfortunately, this work is but little known in England, and it is one of my objects to set it forth. The complicated sequence of phenomena known as the "reaction to cold" includes very remarkable changes in innervation, and in my judgment Professor Winternitz does not exaggerate when he states that heat (plus or minus to the body) may, according to its degree and the duration and application (local or otherwise), strengthen, suspend or alter the character of innervation, local or general.

At present many such conclusions, which appear to be well founded, await substantiation or modification by independent hydrological research in this country.—Yours faithfully,

R. FORTESQUE FOX, M.D.

36 Devonshire Place, W.,

May 30, 1914.

